# Cold-Active *Shewanella glacialimarina* TZS-4_T_ nov. Features a Temperature-Dependent Fatty Acid Profile and Putative Sialic Acid Metabolism

**DOI:** 10.3389/fmicb.2021.737641

**Published:** 2021-10-01

**Authors:** Muhammad Suleman Qasim, Mirka Lampi, Minna-Maria K. Heinonen, Berta Garrido-Zabala, Dennis H. Bamford, Reijo Käkelä, Elina Roine, Leif Peter Sarin

**Affiliations:** ^1^ RNAcious Laboratory, Molecular and Integrative Biosciences Research Programme, Faculty of Biological and Environmental Sciences, University of Helsinki, Helsinki, Finland; ^2^ Doctoral Programme in Microbiology and Biotechnology, University of Helsinki, Helsinki, Finland; ^3^ Molecular and Integrative Biosciences Research Programme, Faculty of Biological and Environmental Sciences, University of Helsinki, Helsinki, Finland; ^4^ Helsinki University Lipidomics Unit HiLIPID, Helsinki Institute of Life Science HiLIFE and Biocenter Finland, Helsinki, Finland; ^5^ The Laboratory of Structural Biology, Helsinki Institute of Life Science HiLIFE, Helsinki, Finland

**Keywords:** *Shewanella*, cold-active bacteria, sea ice, sialic acid metabolism, polyunsaturated (essential) fatty acids

## Abstract

Species of genus *Shewanella* are among the most frequently identified psychrotrophic bacteria. Here, we have studied the cellular properties, growth dynamics, and stress conditions of cold-active *Shewanella* strain #4, which was previously isolated from Baltic Sea ice. The cells are rod-shaped of ~2μm in length and 0.5μm in diameter, and they grow between 0 and 25°C, with an optimum at 15°C. The bacterium grows at a wide range of conditions, including 0.5–5.5% w/v NaCl (optimum 0.5–2% w/v NaCl), pH 5.5–10 (optimum pH 7.0), and up to 1mM hydrogen peroxide. In keeping with its adaptation to cold habitats, some polyunsaturated fatty acids, such as stearidonic acid (18:4n-3), eicosatetraenoic acid (20:4n-3), and eicosapentaenoic acid (20:5n-3), are produced at a higher level at low temperature. The genome is 4,456kb in size and has a GC content of 41.12%. Uniquely, strain #4 possesses genes for sialic acid metabolism and utilizes *N*-acetyl neuraminic acid as a carbon source. Interestingly, it also encodes for cytochrome c3 genes, which are known to facilitate environmental adaptation, including elevated temperatures and exposure to UV radiation. Phylogenetic analysis based on a consensus sequence of the seven 16S rRNA genes indicated that strain #4 belongs to genus *Shewanella*, closely associated with *Shewanella aestuarii* with a ~97% similarity, but with a low DNA–DNA hybridization (DDH) level of ~21%. However, average nucleotide identity (ANI) analysis defines strain #4 as a separate *Shewanella* species (ANI score=76). Further phylogenetic analysis based on the 92 most conserved genes places *Shewanella* strain #4 into a distinct phylogenetic clade with other cold-active marine *Shewanella* species. Considering the phylogenetic, phenotypic, and molecular characterization, we conclude that *Shewanella* strain #4 is a novel species and name it *Shewanella glacialimarina* sp. nov. TZS-4_T_, where *glacialimarina* means sea ice. Consequently, *S. glacialimarina* TZS-4_T_ constitutes a promising model for studying transcriptional and translational regulation of cold-active metabolism.

## Introduction

The genus *Shewanella* belongs to the order *Alteromonadales* of class *Gammaproteobacteria*. In 1985, MacDonell and Colwell (1985) first characterized members of this group comprising Gram stain negative, rod-shaped, and facultative anaerobic bacteria, and in 2004, they were classified into family *Shewanellaceae* (Ivanova et al., 2004). Members of this family have been isolated from diverse aquatic and marine habitats, including deep sea, ocean sediments, freshwater, and sea ice (Li et al., 2014; Luhtanen et al., 2014; Kim et al., 2016; Wang and Sun, 2016). *Shewanella* species are known to be active in a broad temperature range, comprising both psychrotrophic (*Shewanella baltica*, *Shewanella frigidimarina*, and *Shewanella polaris*) and mesophilic (*Shewanella oneidensis*, *Shewanella algae*, and *Shewanella putrefaciens*) bacteria (Rossello-Mora et al., 1995; Khashe and Janda, 1998; Venkateswaran et al., 1999; Bozal et al., 2002; Vogel et al., 2005; Cha et al., 2020). *Shewanella* bacteria are known for their versatile metabolic pathways and wide range of electron acceptors, including various oxidized metals, such as Mn(III), Mn(IV), Fe(III), U(VI) (Myers and Nealson, 1988; Nealson and Saffarini, 1994; Fredrickson et al., 2000), as well as nitrate, sulfite, thiosulfate, and elemental sulfur (Fredrickson et al., 2008), which are vital for bioremediation and biogeochemical cycles.

*Shewanella* spp. are saprophytic and form an integral part of the marine microflora. Certain *Shewanella* species, such as *S. putrefaciens* and *S. baltica*, have been associated with spoilage of fishery food products due to their psychrotrophic nature, which permits growth at low temperatures (Jørgensen and Huss, 1989; Vogel et al., 2005). These bacteria reduce trimethyl-amine-*N*-oxide (TMAO) to trimethylamine (TMA), which generates a pungent odor that alongside hydrogen sulfide (H_2_S) gas, produced by degradation of amino acids, further exacerbates the spoilage process (Gram et al., 1987). Recent emergence of antibiotic resistance and auxiliary metabolic pathways, such as protein and lipid degradation, may further benefit some food spoilage species of *Shewanella*, resulting in additional cold storage problems.

*Shewanella* strain #4 was isolated from Baltic Sea ice around Tvärminne Zoological Station in Hanko, Finland (Luhtanen et al., 2014). Phylogenetic analysis of *Shewanella* strain #4 revealed a distinct clade of cold-active *Shewanella* species, including *S. frigidimarina*, *S. polaris*, and *S. arctica* (Bozal et al., 2002; Kim et al., 2012; Cha et al., 2020). Its ability to thrive in cold, icy conditions and its association with marine life makes *Shewanella* strain #4, a suitable candidate to further study the metabolic and gene expression changes in bacteria adapted to cold environments. For example, we located a complete gene cluster associated with sialic acid metabolism and showed that strain #4 can metabolize sialic acid as a carbon source – a characteristic that is generally found in *Vibrio choleora*, *Yersinia pestis*, *Clostridium perfringens*, etc., and regarded as advantageous in commensal and pathogenic bacteria. Based on our biochemical, physiological, and phylogenetic results, we propose *Shewanella* strain #4 as a novel *Shewanella* species named *Shewanella glacialimarina* TZS-4_T_.

## Materials and Methods

### Bacterial Strains and Isolation

The bacterial strain #4 (hereafter referred to as *S. glacialimarina* TZS-4_T_) was previously isolated from a Baltic Sea ice sample (Luhtanen et al., 2014). Two other *Shewanella* strains, *S. frigidimarina* ACAM 591 and *S. baltica* LMG 2250, were purchased from the Leibniz Institute DSMZ-German collection of microorganisms under catalog number DSM-12253 and DSM-9439, respectively. All strains were grown on a solid medium containing rich 25% w/v marine broth [abbreviated as rMB; 7.5g peptone (Sigma-Aldrich), 1.5g yeast extract (Fisher Bioreagents), and 9.35g marine broth (BD-Difco) in 1L of ddH_2_O (Milli-Q, Merck)] agar (15g/L) and stored in 30% v/v glycerol stock at −80°C.

### General Characterization of Bacteria

#### Gram Staining

A glass slide fixed with bacterial cells was first stained with crystal violet dye solution (Sigma) (2% w/v crystal violet dye in 95% v/v ethanol) for Gram staining. These slides were flooded with Gram’s iodine [1g iodine (Riedel-de Haen) and 2g potassium iodide (Merck) in 300ml distilled water] and counterstained with safranin (Merck) (2.5% w/v in 95% v/v ethanol; Beveridge, 2001). Samples were observed under a microscope at 100× and 1,000× magnification.

#### Growth Conditions

The cells were cultured on rMB agar. After 48h of growth, a single colony of *S. glacialimarina* TZS-4_T_ was transferred to 50ml of rMB and grown for 48h at 15°C with constant aeration at 200rpm. Fresh rMB was inoculated with the starter culture to an optical density at 600nm (OD_600_) of 0.2, and the cells were grown until the desired OD_600_ was reached. The turbidity was measured using an Eppendorf Biophotometer.

#### Hemolysis, Motility, and Hydrogen Sulfide Production Test

Blood agar plates (3% v/v sheep blood with agar base no.2) (Labema) were used to check hemolytic activity. Iron agar test was performed to determine bacterial motility and H_2_S production (Gram et al., 1987). Both blood agar and iron agar tubes were inoculated with the starter culture and incubated at 15°C for 72h.

#### Catalase and Oxidase Test

Catalase enzyme activity was confirmed with 3% v/v hydrogen peroxide (H_2_O_2_) (Fisher Chemical) (Taylor and Achanzar, 1972), and oxidase reagent (1% w/v tetramethyl-*p*-phenylenediamine dihydrochloride) (Acros Organics) was used for the oxidase test (Kovacs, 1956). Both catalase and oxidase test were done according to established protocols.

#### Temperature, pH, and Salinity Conditions

The cells were prepared in rMB media as described in section Growth Conditions, except for the starter culture to assess growth at 0°C, which was grown at 4°C for 48h (necessary for growth at 0°C). Various growth conditions, namely, temperature (0, 4, 15, and 25°C), pH (4.5–10.5), salinity (5–55g/L NaCl), and hydrogen peroxide (0.5–4mM)-induced oxidative stress, were evaluated as a time-course series by determining the change in OD_600_ values.

#### Carbon Assimilation Using Minimal Growth Media

Carbohydrate assimilation was determined by growing *S. glacialimarina* TZS-4_T_ on M-9 minimal media [64.0g Na_2_HPO_4_, 15.0g KH_2_PO_4_ (Acros Organics), 2.5g NaCl (Fisher Chemical), and 5.0g NH_4_Cl (Fisher Chemical) in 1L ddH_2_O (Milli-Q) to make 5× stock] in the presence of a sole carbon source, namely, 200mM glucose (VWR Chemicals), fructose (Fisher Chemical), maltose (Alfa Aesar), or galactose (Fluka), respectively. The cells were grown, as described in section Growth Conditions, in 10ml of M-9 minimal media using starter cultures grown in rMB. Bacterial growth was monitored by measuring OD_600_. M-9 media without any carbon source was used as negative control.

#### Ammonia Production

Ammonia production was analyzed by colorimetric assays using Nessler’s reagent (VWR Chemicals). The cells were grown in 50ml rMB for 24 or 48h, as described in section Growth Conditions. Cells were removed by centrifugation for 5min at 3200*g*, 4°C, after which 10ml of supernatant was collected. Subsequently, 1ml of Nessler’s reagent was added to the supernatant and the formation of a dark blue color indicating the presence of ammonia.

### Phenotypic Characterization Using Biolog GENIII Plates

Biolog GENIII microplates were used for initial phenotypic characterization, providing a standardized platform to test 94 different phenotypic characteristics, including 71 carbon utilization assays and 23 chemical sensitivity tests. Samples were prepared and tested according to the manufacturer’s instructions. All assays were performed in triplicates.

### Antibiotic Resistance

The bacteria were grown in both rMB media and rMB agar in the presence of different antibiotics, such as ampicillin (100μg/ml in ddH_2_O), kanamycin (50μg/ml in ddH_2_O), tetracycline (10μg/ml in ddH_2_O), and chloramphenicol (25μg/ml in 99.5% EtOH). The growth on antibiotic media was observed after 24h, and results were interpreted as resistant (+) or susceptible (−).

### Genome Sequencing and Phylogenetic Analysis of *Shewanella*

The genomic sequence of *S. glacialimarina* TZS-4_T_ was determined using the Pacific Biosciences PacBio Sequel sequencing technology at the DNA Sequencing and Genomics Laboratory (Helsinki Institute of Life Science, University of Helsinki). Phylogenetic trees based on maximum likelihood, neighbor-joining, and maximum parsimony methods using the consensus sequence of the seven gene copies of 16S rRNA were constructed on MEGA software with 100 replicates as bootstrap value. To infer genome-based phylogeny, we constructed a concatenated bacterial core-gene set phylogenetic tree by the up-to-date bacterial core-gene set (UBCG) method (Na et al., 2018). A model phylogenetic tree was depicted for species delineation based on average nucleotide identities (ANI) score using the fastANI (ver. 1.31) and OrthoANI tools (ver. 0.90; Lee et al., 2016; Jain et al., 2018). OrthoANI calculated the ANI score of query organism against a group of reference genomes and generated a distance matrix. The phylogenetic tree with its heat-map shows the relationship among the genome sets. We used AAI-profiler^1^ (Medlar et al., 2018) as an additional method to predict the taxonomic identity of the *S. glacialimarina* TZS-4_T_. Open reading frames were predicted using GenMark.hmm prokaryotic version 3.25 (Besemer, 2001) applying the same search parameters as used for *S. frigidimarina* NCIMB400. The genome sequence has been submitted to GenBank under the accession number CP041216.

### Transmission Electron Microscopy

The size and shape of *S. glacialimarina* TZS-4_T_ were determined by transmission electron microscopy (TEM). The cells were grown in rMB media at 15°C until they reached an OD_600_ of 0.6, which corresponds to approximately 10^8^ cells/ml. Later, 2μl of the culture was incubated on carbon-coated Cu mesh grids for 1min and then negative stained with filtered, neutral 2% uranyl acetate for 15s. TEM examination was conducted using a JEOL JEM-1400 microscope (Jeol Ltd., Tokyo, Japan) operating at 80kV and equipped with Gatan Orius SC 1000B bottom-mounted CCD camera (Gatan Inc., United States).

### Scanning Electron Microscopy

For scanning electron microscopy (SEM), cells were grown to OD_600_ of 0.6, as described in section Growth Conditions. Cells were harvested from 10ml of culture by centrifugation (5min, 3,200*g*, 4°C) and resuspended in the original volume with phosphate buffer saline (PBS). Cells were fixed with 2.5% v/v glutaraldehyde at 4°C for 20h. After fixation, the cells were washed twice with PBS using centrifugation and resuspended in half of the original volume of PBS. Cell suspension (100μl) was spread on concanavalin A-coated glasses, fixed with osmium tetroxide, and dehydrated through ethanol series (50, 70, and 96%). Dehydrated cells were incubated in hexamethyldisilazane overnight and coated with platinum. Finally, SEM examination was conducted using FEI Quanta 250 Field Emission Gun (FEG) Scanning Electron Microscope.

### Fatty Acid Composition

Starter culture of *S. glacialimarina* TZS-4_T_ was used to inoculate a 50ml culture as described in section Growth Conditions and grown until it reached OD_600_ of 0.8. Samples were prepared at different temperature conditions, i.e., 5, 15, and 25°C, and the pellet from 5ml culture was washed five times with PBS. Finally, the washed pellet was used for the analysis of fatty acid (FA) composition by gas chromatography. The bacterial pellets were subjected to transmethylation by heating with 1% v/v H_2_SO_4_ in methanol under nitrogen, and the formed FA methyl esters were extracted with hexane (Christie, 1993). The samples were dried with anhydrous Na_2_SO_4_ and concentrated. The FA methyl esters were identified based on their mass spectra recorded by GCMS-QP2010 Ultra (Shimadzu Scientific Instruments, Kyoto, Japan) and compared to the spectra of several authentic standard mixtures (including Supelco 47,080-U Bacterial Acid Methyl Ester BAME Mix) and published reference mass spectra (Christie, 2019). Quantitative composition was determined by using a Shimadzu GC-2010 Plus with flame ionization detector. The responses were corrected by using the theoretical correction factors for this detector (Ackman, 2007). Both gas chromatographs were equipped with a Zebron ZB-wax capillary column (30m, 0.25mm ID and film thickness 0.25μm; Phenomenex, Torrance, CA, United States). The FA compositions are expressed as mol% profiles, and the FAs are marked using the following abbreviations: (carbon number):(number of double bonds) n-(position of the first double bond calculated from the methyl end) (e.g., 16:1n-7 for palmitoleate). The identified *iso* and *anteiso* branches and the 3-OH substituents of the FAs were indicated. The FA components exceeding 0.1mol% at least in one sample were listed, and the remaining were summed as trace FAs. The fold change analysis of unsaturated FA relative concentrations was performed w.r.t 15°C samples as reference. SD was calculated using propagation of error.

### RNA Isolation

RNA isolation was performed using acidic phenol:bromochloropropane (BCP; Chomczynski and Mackey, 1995; Gregorova et al., 2020). The cells were grown from the starter culture in 50ml of rMB until it reached OD_600_ of 0.8. The cells were harvested at 3200*g*, 4°C for 10min and stored in −80°C. The pellet was resuspended in 4ml of 0.9% w/v NaCl solution followed by 4ml of acidic phenol (Sigma) and 800μl of BCP (Acros organic). Glass beads were added to break the bacterial cells and vortexed for 10min at room temperature (RT). The lysate was centrifuged at 10000*g* for 10min at RT, and aqueous phase was collected. The aqueous phase was re-extracted twice with phenol:BCP (2ml of acidic phenol and 400μl of BCP). Total RNA from the aqueous phase was precipitated by adding 2.5 vol of 99.6% v/v EtOH, at −20°C overnight and pelleted by centrifugation at 10000*g* for 20min at 4°C. The RNA pellets were air-dried and resuspended in RNAase-free ddH_2_O water. RNA concentration was measured using a Nanodrop 2000 spectrophotometer (Thermo Scientific) and run on 2% w/v agarose, Tris-borate EDTA (TBE) gel with of Midori green (4μl per 100ml of 2% agarose) for quality assessment. The images were captured on Gel-doc XR (Biorad).

### Reverse Transcription-Quantitative PCR

Primers were designed for each gene using the IDT PrimerQuest tool^2^ (Supplementary Table ST-1). The purified RNA (30μg) was DNAase treated with 7U of RQ1 RNAase-Free DNase (Promega). cDNA was synthesized using 200U of Maxima reverse transcriptase primed with 1μl random hexamers (0.2μg/μl) (Thermo Scientific) and 3μg of DNase-treated RNA. Both DNAase treatment and reverse transcription reactions were performed according to the manufacturers’ instructions. cDNA synthesis was validated by PCR (using primers listed in Supplementary Table ST-1), and the products were analyzed on a 2% agarose gel (Supplementary Figure SF-1). Quantitative PCR reactions were performed using Perfecta SYBR green FastMix, Low ROX (Quantabio) consisting of 5μl of 2× Perfecta mix, 0.4μl of 2μM primer mix (fwd and rev primers), adjusted to 10μl of final volume with ddH_2_O water. Control samples were included to check amplification arising from contaminating genomic DNA and from the primer-dimer formation. All the samples were run in technical triplicates on Quantstudio 3 Real-time PCR system (Thermo Fisher Scientific). The transcript was amplified using the following conditions: 95°C for 3min followed by 50cycles of 95°C for 30s, 62°C for 30s, and 72°C for 30s. A melting curve profile was generated to determine the formation of a single amplification product. Primer efficiency for all the genes was determined using the formula Efficiency (%)=100×(10^-1/slope-1^) with 5-fold dilution series of template cDNA. Comparison of fold change between different target genes was using ∆∆C_T_ method, and gyrase A (*gyrA*) gene was selected as reference for normalization (Livak and Schmittgen, 2001). Data analysis and statistical tests for RT-qPCR were performed on GraphPad Prism 9.

### PCR and Agarose Gel Electrophoresis

The RT-qPCR primers (Supplementary Table ST-1; housekeeping genes *gyrA*, 16S rRNA, and *recA*, and target genes *nanA* and *nanH*) were tested on cDNA using end-point PCR. The PCR reaction conditions were 95°C for 2min followed by 35cycles of 95°C for 30s, 62°C for 30s and 72°C for 30s. The amplification temperature of the primer pairs was confirmed using gradient PCR from 60 to 64°C (data not shown). The products of PCR reactions were analyzed on a 2% agarose TBE gel. The images were captured using a Gel-doc XR (Biorad).

### Nucleotide Sequence Accession Numbers

GenBank accession numbers of *Shewanella* whole genome sequences used for fastANI and UBCG analysis were as follows: NZ_CP050313.1, NZ_CP047422.1, NZ_CP018456.1, NZ_CP033575.1, NZ_CP046378.1, NC_008700.1, NC_017571.1, NC_017579.1, NC_009052.1, NC_009665.1, NC_009997.1, NC_011663.1, NC_016901.1, NZ_CP028730.1, NZ_CP028355.1, NZ_LS483452.1, NZ_CP022358.1, NZ_CP045857.1, NC_007954.1, NZ_CP041783.1, NC_008345.1, NC_010334.1, NZ_CP020472.1, NZ_CP020373.1, NZ_CP034015.1, NC_009092.1, NZ_CP041153.1, NZ_CP022272.1, NZ_CP036200.1, NC_004347.2, NC_009901.1, NC_011566.1, NZ_CP041036.1, NZ_CP014782.1, NC_017566.1, NC_009438.1, NZ_CP046329.1, NZ_LR134321.1, NZ_LR134303.1, NZ_CP028435.1, NC_009831.1, NC_008577.1, NZ_CP048031.1, NZ_CP022089.2, NZ_CP039928.1, NC_008321.1, NC_008322.1, NZ_CP015194.1, NZ_CP041329.1, NZ_CP041151.1, NC_008750.1, NZ_CP023019.1, NZ_CP041614.1, NC_014012.1, and NC_010506.1.

## Results

### *Shewanella glacialimarina* TZS-4_T_ Grows Well in Enriched Marine Broth

Bacterial strain *S. glacialimarina* TZS-4_T_ was originally isolated from the Baltic Sea ice outside of Tvärminne Zoological station, Hanko, Finland, and cultivated on Zobell media containing undefined Baltic Sea water (Luhtanen et al., 2014). To standardize the growth media and remove any influence of seasonal fluctuation in seawater composition, we investigated if marine broth (MB) could be used to cultivate *S. glacialimarina* TZS-4_T_. First, the salt concentration of the Baltic Sea water used was determined to be 6.9g/L, which is consistent with brackish coastal water. We then compared the growth in Zobell media and in rMB, which has a defined composition with a total salt concentration of ~7.8g/L. rMB was found to be well suited for *S. glacialimarina* TZS-4_T_ and supports a faster growth rate and a higher cell density at stationary phase (Supplementary Figure SF-2). Based on the favorable growth characteristics, rMB was adopted for all subsequent experiments in this study.

### Phylogenetic Analysis and Average Nucleotide Identity Distinguishes *S. glacialimarina* TZS-4_T_ as a Distinct *Shewanella* Species

**Figure 1:**
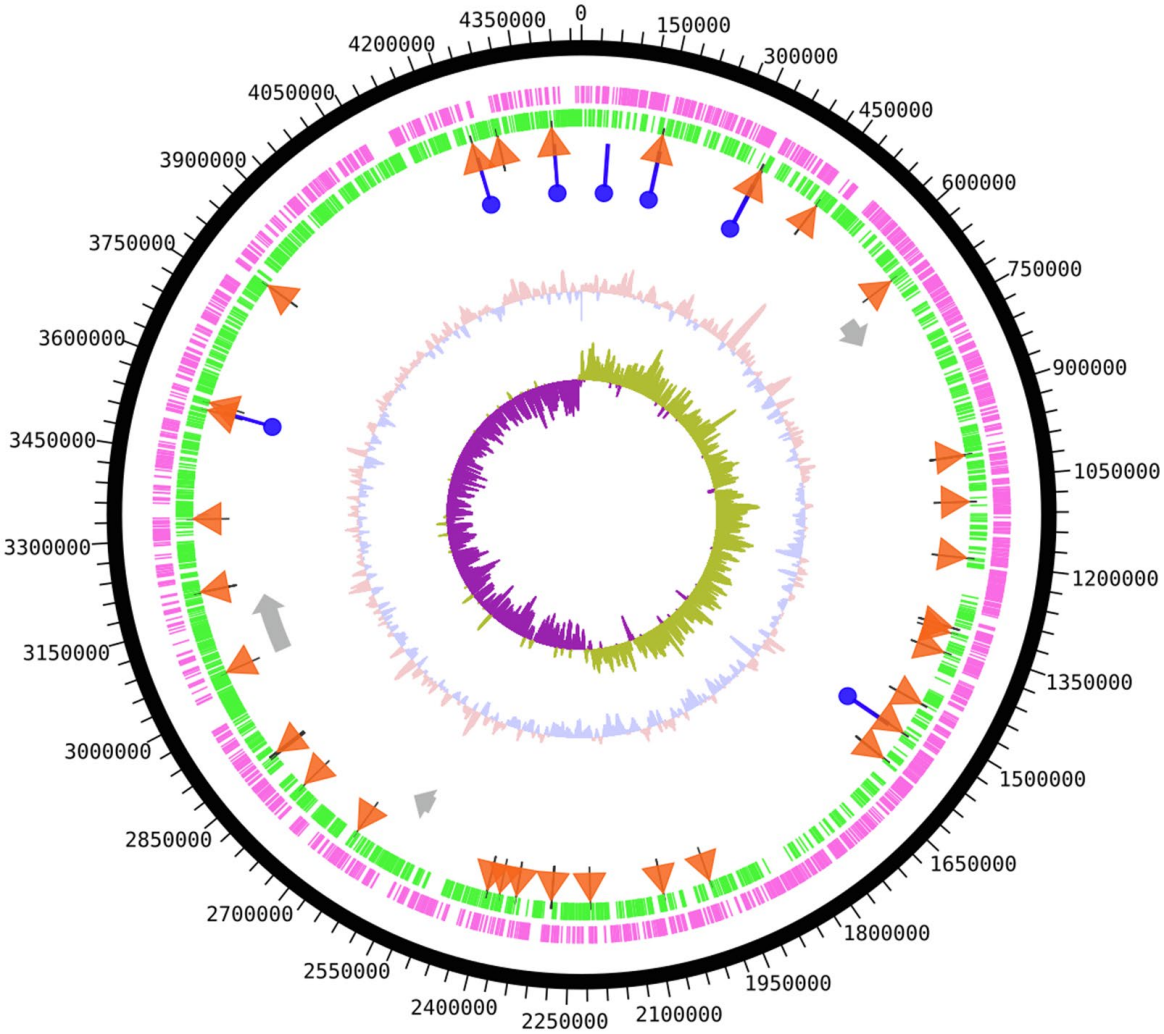
Genome map of *Shewanella glacialimarina* TZS-4_T_. Circular map from outside to center; coding DNA sequence (CDS) forward strand and CDS reverse strand are shown in magenta and green, respectively. tRNA genes are depicted as orange triangles and rRNA genes as blue circles. Prophage gene segments are represented as grey arrows. The innermost two segments denote GC content (salmon/blue) and GC skew (purple/olive) around the chromosome. The map was produced using the DNAplotter tool on Artemis (Carver et al., 2012).

Genomic DNA sequencing of *S. glacialimarina* TZS-4_T_ was performed on a PacBio Sequel platform and assembled using hierarchical genome-assembly process (HGAP) analysis (Chin et al., 2013), achieving a coverage depth of 90.54×, indicating a high read confidence. The number of PacBio sequence reads was 57,211 with a N50 read length of 16,906bp. Its genome comprises a single circular chromosome of 4,456kb with a GC content of 41.12%, featuring 3,906 predicted open reading frames (ORFs) with 3,725 protein-coding sequences (Figure 1).^3^ There are a total of 118 RNA coding genes, 91 of which encode for tRNA and 22 for rRNA, of which eight encode for 5S rRNA, seven for 16S rRNA, and seven for 23S rRNA. In addition, two complete and one incomplete prophage segments were identified (Figure 1) by phispy tool on the RAST server and PHASTER (Arndt et al., 2016) with hits to predicted proteins of, e.g., phage 1/44, which was previously reported to infect the cold-active *Shewanella* sp. #44 strain (Luhtanen et al., 2014; Senčilo et al., 2015).

**Figure 2:**
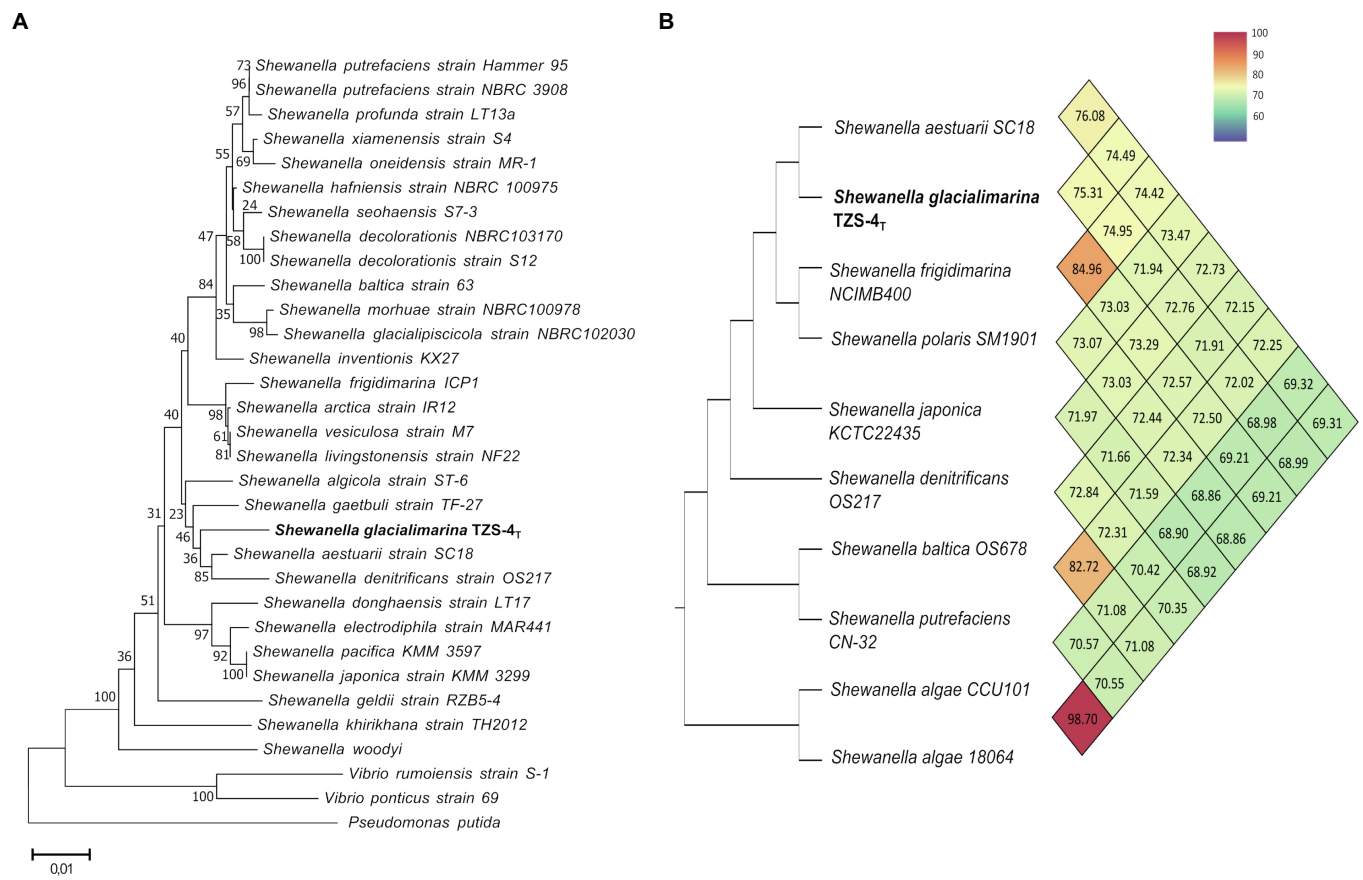
Phylogenetic analysis of *S. glacialimarina* TZS-4_T_. **(A)** Phylogenetic tree based on Maximum Likelihood tree construction of 16S rRNA sequences with 100 replicates as bootstrap. The taxonomic position of *S. glacialimarina* TZS-4_T_ (bolded) alongside other closely related strains of genus *Shewanella*. *Pseudomonas putida* is shown as an outgroup at the end of the tree. **(B)** Phylogenetic tree constructed by the orthroANI tool showing the position of *S. glacialimarina* TZS-4_T_ and nine closely related *Shewanella* species. ANI score matrix is represented as a heat map from blue (low score) to red (high score).

Next, we wanted to position *S. glacialimarina* TZS-4_T_ in regard to other *Shewanella* species. To this end, we constructed phylogenetic trees based on the 16S rRNA gene by maximum likelihood, neighbor-joining, and maximum parsimony methods with 100 replicates as bootstrapping and compared the tree topologies. The trees included 29 *Shewanella* strains representing 13 different phenotypic sub-groups with *Vibrio* and *Pseudomonas* strains as out-groups. The phylogenetic analyses yielded equivalent results (Supplementary Figure SF-3), with maximum likelihood showing that *S. glacialimarina* TZS-4_T_ forms a differentiated branch with *S. aestuarii* SC18 (97.25%) and *S. denitrificans* OS217 (96.53%) branching next to it (Figure 2A). Similar results were obtained using AAI-profiler which compares the predicted proteome of an organism to Uniprot database (Supplementary Figure SF-4; Medlar et al., 2018). Limiting the phylogenetic analysis to the 92 most conserved genes yields a similar distribution, with cold-active Antarctic and Arctic Sea bacteria, such as *Shewanella* sp. Arc9-LZ, *S. aestuarii* SC18, *S. polaris*, and *S. frigidimarina* clustering alongside *S. glacialimarina* TZS-4_T_ (Supplementary Figure SF-5).

To obtain independent confirmation, two bioinformatic tools – fastANI and orthroANI – were used to calculate the average nucleotide identity (ANI) values for *S. glacialimarina* TZS-4_T_ in relation to other *Shewanella* species. For fastANI analysis, the comparison was made against whole genome sequences (WGS) of 55 *Shewanella* strains available in NCBI. The result showed that *S. aestuarii* yielded the highest ANI score with 611 matching sequences *S. glacialimarina* TZS-4_T_, followed by *S*. Arc9-Lz and *S. frigidimarina* (Supplementary Table ST-2). Further comparison was done using orthroANI on a limited subset of nine closely related *Shewanella* species, which delineated *S. glacialimarina* TZS-4_T_ (ANI=75.31) along with *S. aestuarii* (ANI=76.08; Figure 2B). The ANI scores derived from fastANI and orthroANI are both below 95%, which is currently considered as the benchmark value for classifying bacterial species (Richter and Rosselló-Móra, 2009). Taken together, this implies that *S. glacialimarina* TZS-4_T_ is genetically divergent and constitutes a distinct *Shewanella* species, which clusters with other cold-active marine *Shewanella* species.

### Phenotypic Characterization of *S. glacialimarina* TZS-4_T_ Uncovers a Resilient, Cold-Active Environmental Bacterium

To determine the characteristics of *S. glacialimarina* TZS-4_T_, we grew the bacteria on rMB-agar where they form smooth, circular, and convex colonies with a diameter of 1–2mm and a milky white appearance. Gram staining confirmed that *S. glacialimarina* TZS-4_T_ is a Gram-negative, rod-shaped bacterium. Interestingly, after 3days of growth the colonies turn reddish-brown. A similar phenomenon was observed when the bacteria were grown for up to 29h in rMB-broth at 15 and 25°C (Supplementary Figure SF-6). We also found that pigment formation is not affected by light intensity (data not shown), but the cells are less pigmented at low growth temperatures (Supplementary Figure SF-6). This suggests that the pigment may have a role in temperature or UV-radiation adaptation, as was previously proposed for a *S. frigidimarina* strain isolated from Antarctic glacier snow (Martin-Cerezo et al., 2015), where the red pigment in question was identified as cytochrome c3. Indeed, a closer inspection of the *S. glacialimarina* TZS-4_T_ genome revealed genes encoding for a putative cytochrome c3 family protein.

**Figure 3:**
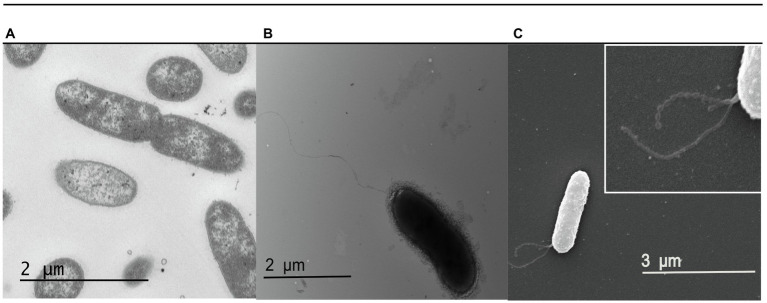
Electron microscopy visualization of *S. glacialimarina* TZS-4_T_. **(A,B)** Cells visualized with TEM after negative staining with 2% uranyl acetate. **(C)** Cells visualized with SEM. The insert (white box at the top right) shows a close-up of the nanowire-like structures originating from the outer membrane of the bacteria.

Next, we examined the physiology of *S. glacialimarina* TZS-4_T_ using transmission electron microscopy (TEM). This showed a rod-shaped bacterium 0.5μm in diameter and ~2μm in length (Figure 3A) with clearly visible long polar flagella (Figure 3B). We also observed various bead-shaped extensions surrounding the bacteria. To visualize these, SEM analysis was performed, exposing a granular structure extending from the outer membrane of the bacteria (Figure 3C; highlighted in the insert). These extensions resemble a previously identified nanowire-like structure in *S. oneidensis* MR-1 (Gorby et al., 2006). The nanowires function as channels for extracellular electron transport in bacterial communities, allowing electron transfer from external surfaces, such as oxidized metals (Pirbadian et al., 2014). Genes encoding for nanowire-like structures, such as *omcA*/*mtrC*, were also identified in the *S. glacialimarina* TZS-4_T_ genome, but the potential function of these structures is unclear and remains to be determined.

To further study the phenotypic characteristics of *S. glacialimarina* TZS-4_T_, we performed a number of standardized panel tests, comparing the results to the related psychrophilic bacterium *S. frigidimarina* (type strain ACAM 591) and the mesophilic reference bacterium *S. baltica* (type strain LMG 2250; Table 1). *Shewanella glacialimarina* TZS-4_T_ is a motile, facultatively anaerobic bacteria that utilizes glucose and maltose as its sole source of carbon. However, the bacterium does not produce H_2_S, which is a characteristic feature of many *Shewanella* strains associated with fish spoilage in cold storage. Furthermore, *S. glacialimarina* TZS-4_T_ is also capable of cytochrome oxidase and catalase production.

**Table 1:** Phenotypic characteristics of *S. glacialimarina* TZS-4_T_ alongside other *Shewanella*-type strains.

Characteristics	*Shewanella baltica* LMG 2250	*Shewanella frigidimarina* ACAM 591	*Shewanella glacialimarina* TZS-4_T_
Size (μm)			
Length	N.D.	1–3.7μm (Bozal et al., 2002)	~2μm
Width	N.D.	0.5μm (Bozal et al., 2002)	0.5μm
Temperature (°C)	5–30	5–25	0^†^–25
Salinity (%)	0.5–5.5	0.5–5.5	0.5–5.5
pH	5.5–9.5	5.5–9.5	5.5–9.5
Growth in minimal media with sole carbon sources			
Glucose	+	+	+
Fructose	−	−	−
Maltose	+	+	+
Galactose	−	−	−
Resistance to antibiotics			
Ampicillin	+	−	+
Kanamycin	−	−	−
Tetracyclin	+	−	−
Chloramphenicol	−	−	−
GC content %	46.25	41.48	41.12
H_2_S gas production	+	−	−
Anaerobic growth	+	+	+
Pigment	Reddish brown	Reddish brown	Salmon
Oxidase	+	+	+
Hemolysis on blood agar	+ (α)	+ (α)	+ (α)
Gelatin hydrolysis	+	+	+
Assimilation of ammonia	+	+	+
Catalase	+	+	+
*N*-acetyl neuraminate lyase activity^*^	N.D.	N.D.	+
Gelatinase activity^*^	N.D.	N.D.	+
Tween-40^*^	N.D.	N.D.	+

+, positive; −, negative; and N.D., not determined.

* Biolog plate results.

† Growth at 0°C only following pre-conditioning (starter culture grown at 4°C).

**Figure 4:**
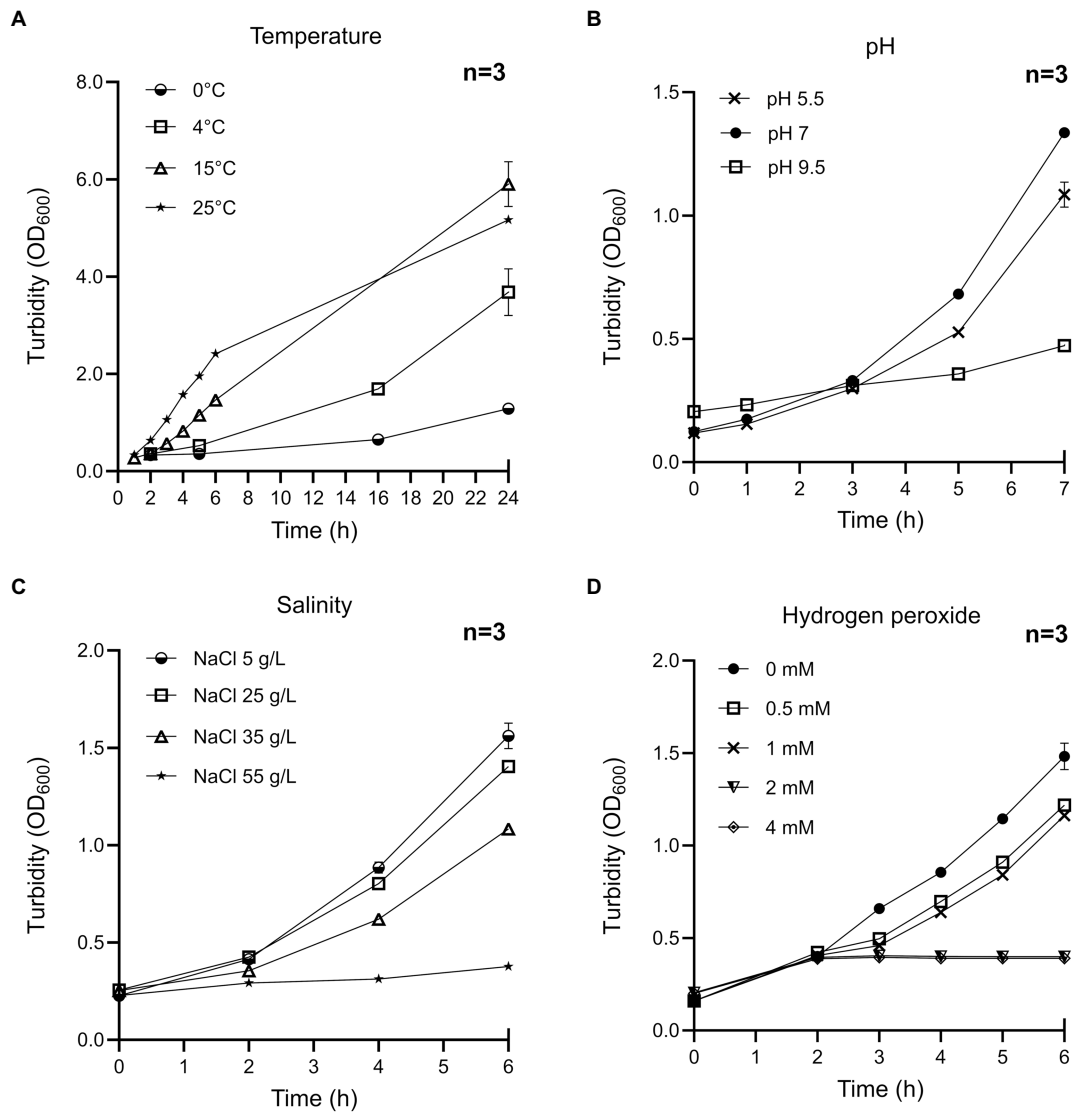
Characterization of growth conditions of *S. glacialimarina* TZS-4_T_ bacteria. Growth curves at different **(A)** temperature, **(B)** pH, **(C)** NaCl, and **(D)** hydrogen peroxide concentration. The error bars denote SD.

Next, to determine the growth rate, we explored different physiological conditions, such as temperature, pH, salinity, and hydrogen peroxide induced oxidative stress. We observed that *S. glacialimarina* TZS-4_T_ is psychrophilic and grows from low to ambient temperatures in a range between 0 and 25°C, with an optimum at 15°C (Figure 4A; Supplementary Figure SF-2). However, growth at 0°C requires preconditioning (growth at 4°C) of the starter cultures, as those grown at 15°C do not cope with the cold-shock and yield no growth at freezing conditions. During the first 6h post-inoculation, the growth rate is the slowest at 0 and 4°C, whereas it is the fastest at 25°C. On the other hand, there is an earlier cessation of growth at high temperature, as the cell density recorded at 24h post-inoculation is lower at 25°C than at 15°C (Figure 4A). Accordingly, at 24h we also observed a lower number of viable cells at 25°C (~1 ×10^9^cfu/ml) than at 15°C (~8 ×10^9^ cfu/ml), and the cells lose their ability to grow altogether at 28°C (data not shown). Indeed, a significant loss of viability is also observed during the 24–48h post-inoculation window (Supplementary Figure SF-2), which is consistent with the culture entering the death phase. *S. glacialimarina* TZS-4_T_ has a propensity for neutral pH conditions with an optimum at pH 7.0, although it tolerates pH ranging from 5.0 to 9.5 (Figure 4B). It also has a high NaCl tolerance, with 35 and 55g/L NaCl reducing growth but not abolishing it (Figure 4C). Since the oxygen solubility from the environment is increased at low temperature (Karbowiak et al., 2009), we analyzed the ability of the cells to tolerate reactive oxygen species by supplementing the growth media with different concentrations of hydrogen peroxide (H_2_O_2_; Figure 4D). The cells grow in the presence of 1mM H_2_O_2_, but 2mM and 4mM H_2_O_2_ concentrations resulted in the complete cessation of growth.

Finally, we investigated the antibiotic resistance of *S. glacialimarina* TZS-4_T_ and found it to be susceptible to chloramphenicol, kanamycin, and tetracycline, but resistant to ampicillin (Table 1). Indeed, sequence analysis revealed that *S. glacialimarina* encodes resistance genes for ampicillin, including class-D β-lactamase and metallo-β-lactamase. Other virulence-associated factors, such as putative T1SS-secreted agglutinin RTX (cytotoxin), putative collagenase genes, and clp proteases, were also identified from the genomic sequence.

### Unsaturated Fatty Acid Production Responds Dynamically to Changes in Temperature

**Figure 5:**
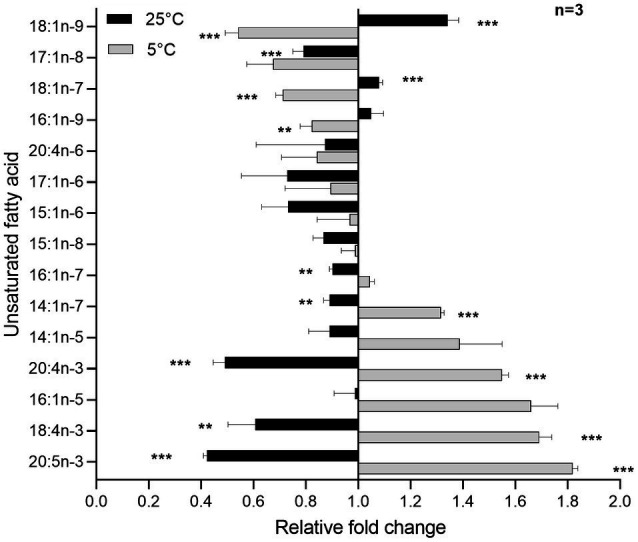
Relative concentrations of unsaturated FAs in *S. glacialimarina* TZS-4_T_ analyzed by GC-FID and described as fold change. Figu re depicts the FA mol% values of *S. glacialimarina* TZS-4_T_ grown to exponential phase (OD_600_=0.8) at 5 and 25°C normalized to the values at 15°C. All FAs are abbreviated as (carbon number):(number of double bonds) n-(position of the first double bond calculated from the methyl end). Error bar shows the SD, Welch *t*-test with Bonferroni correction, two-tailed ^***^ *p* ≤0.001 and ^**^ *p*=0.002 w.r.t 15°C.

To further investigate the cold adaptation of *S. glacialimarina* TZS-4_T_, we analyzed the FA modulation as it is a well-known strategy of cold-active bacteria to maintain membrane fluidity and viability. Our analysis revealed that palmitoleic acid (16:1n-7) was the most prevalent FA component of *S. glacialimarina* TZS-4_T_, constituting 32–37mol% of the total FAs, with the highest values detected at 5°C. In general, *iso*-13:0 and *iso*-15:0 accounted for more than 10mol% and were followed by 12:0, 14:0, and 16:0 that constituted close to 5% (Supplementary Table ST-3). A significant increase of certain unsaturated FAs, including 7-tetradecenoic acid (14:1n-7), eicosatetraenoic acid (20:4n-3), stearidonic acid (18:4n-3), and eicosapentaenoic acid (20:5n-3), was observed at low temperatures (Figure 5). Indeed, at 5°C the ratio of 20:5n-3 had raised close to 6mol%. A previous study on *S. electrodiphila* MAR441 showed similar temperature-dependent regulation of the relative concentration of certain n-3 FAs that have been associated with improved membrane fluidity and increased cell viability at low temperatures (Zhang and Burgess, 2017).

### Sialic Acid Metabolic Genes Are Expressed in *S. glacialimarina* TZS-4_T_

**Figure 6:**
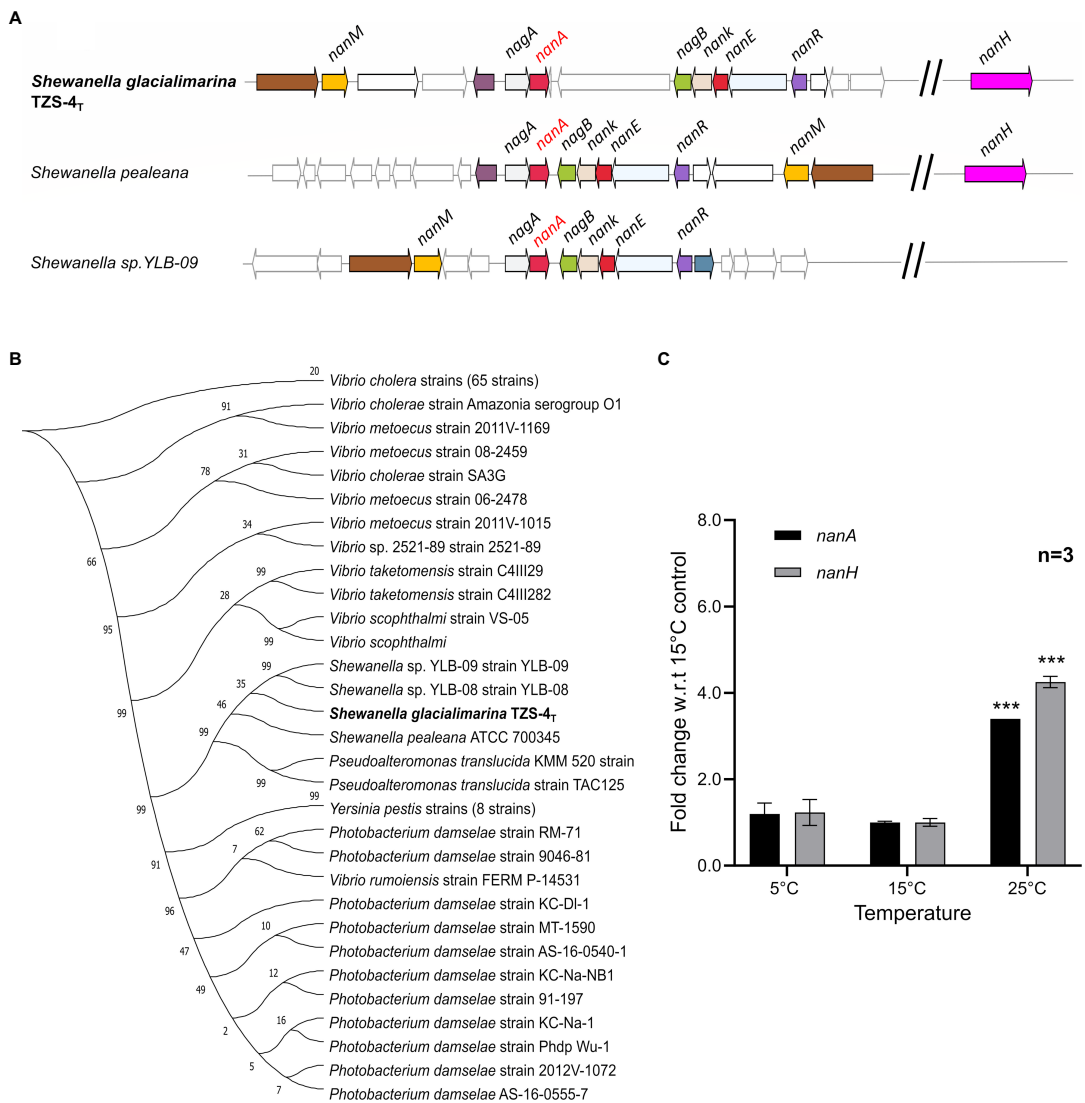
**(A)** Sialic acid metabolism gene cluster in *S. glacialimarina* TZS-4_T_, *Shewanella pealeana,* and *Shewanella* sp. YLB-09. Genes involved in the sialic acid metabolism shown in different colors located around a central *N*-acetylneuraminate lysase (*nanA*) colored in red. **(B)** Phylogenetic tree of *N*-acetyl neuraminate lyase (*nanA*) from *S. glacialimarina* TZS-4_T_ and closely related *nanA* genes from other bacterial species. The sequences were aligned using ClustalW tool, and tree was constructed using 100 replicates as bootstrapping value on MEGA X software. **(C)** Expression analysis by RT-qPCR of sialic acid catabolic enzymes genes in *S. glacialimarina* TZS-4_T_ at different temperatures. SD is shown by the error bars, independent *t*-test, two-tailed ^***^ *p* ≤0.001 w.r.t 15°C.

Interestingly, *S. glacialimarina* TZS-4_T_ features a complete sialic acid metabolism gene cluster in its chromosome, including *nanA* that encodes for the major catalytic enzyme *N*-acetylneuraminate lyase and the sialidase encoding gene *nanH*. Upon blastn search, two distantly related *Shewanella* species, *S. pealeana* and *Shewanella* sp. YLB-09, also contain a similar sialic acid metabolism gene cluster (Figure 6A). Phylogenetic comparison using *nanA* revealed that among *Shewanella*, only these species carry this gene cluster, which is otherwise commonly found among pathogenic bacteria, such as *Vibrio cholerae* and *Yersinia pestis* (Figure 6B). Since other *Shewanella* species, such as *S. putrefaciens*, have been reported to cause serious health disorders among freshwater fish (Paździor et al., 2019), we hypothesize that the sialic acid metabolism gene cluster may play a role as a putative pathogenicity factor in *S. glacialimarina* TZS-4_T_. To determine if the *nanA* and *nanH* genes are transcribed at normal growth conditions, we performed an RT-qPCR expression analysis at 5, 15, and 25°C. Our results show that *nanA* and *nanH* are stably expressed at 5 and 15°C, although a significant upregulation occurs at 25°C w.r.t 15°C (Figure 6C). Furthermore, *nanA* and *nanH* expression remains stable throughout the exponential growth phase and only a slight, albeit not statistically significant downregulation is observed at the stationary phase for cells grown at 5°C (Supplementary Figure SF-7). This shows that the sialic acid enzymes genes are transcribed in *S. glacialimarina* TZS-4_T_ and there is a significant temperature-dependent expression profile. However, the upregulation seen at 25°C suggests that the sialic acid metabolism gene cluster most likely does not further cold-active growth or a role in food spoilage during cold storage.

## Discussion

Bacteria of the genus *Shewanella*, family *Shewanellaceae*, are capable of inhabiting a wide range of aquatic environments due to their versatile physiological characteristics (Lemaire et al., 2020). The Baltic Sea is one of the largest brackish water basins in the world and is partly covered by ice during the winter months (Granskog et al., 2006). During ice formation, channels containing brine are formed (Granskog et al., 2006), which serve as habitats for primary producers, such as unicellular algae, and heterotrophic bacteria, including *Shewanella* (Kaartokallio et al., 2008). Here, we have further characterized *Shewanella* strain #4, which was previously isolated from a Baltic Sea ice sample (Luhtanen et al., 2014), by sequencing its genome and studying its physiological properties, thereby uncovering it to be a new species that we name *Shewanella glacialimarina* TZS-4_T_. *Shewanella glacialimarina* TZS-4_T_ is cold-active, able to grow in freezing temperatures, and in high salinity (up to 6% NaCl; Table 1; Figure 4). Such conditions are fairly common in the Baltic Sea, where *Shewanella* are most often found at depths corresponding to the oxic/anoxic interface (Brettar et al., 2002; Rak and Wieczorek, 2012), which is mixed annually (Hordoir et al., 2015). Furthermore, the shift toward freezing temperature occurs gradually in the Baltic Sea. This gradual change is crucial for *S. glacialimarina* TZS-4_T_, as it enables the bacteria to adapt to the prevailing environmental conditions. Indeed, we have shown that growth of *S. glacialimarina* TZS-4_T_ does not recover following a sudden decrease in temperature from 15 to 0°C, as only bacteria that are preconditioned, i.e., maintained at 4°C, can survive and grow at 0°C (Table 1). During the cold-adaptation phase, bacteria generally produce cold-shock proteins (*Csp*) and store solutes, such as glycerol, sucrose, or mannitol, which provide both cryoprotection and yield an additional carbon source (Tribelli and López, 2018).

*Shewanella glacialimarina* TZS-4_T_ also has additional strategies to cope with the conditions native to its habitat. This is exemplified by the dynamic changes in unsaturated FA production in response to growth temperature. For the cells grown at low temperatures, we observed large relative increases for 14:1n-7, 20:4n-3, 18:4n-3, and 20:5n-3, whereas the profile was shifted toward relatively long saturated and monounsaturated FAs at high temperatures (Figure 5; Supplementary Table ST-3). Such a temperature-specific regulation has previously been reported to modulate lipid composition of bacterial cell membrane maintaining membrane fluidity and cell viability (Zhang and Burgess, 2017). On the other hand, temperature-regulated sialic acid metabolism also directs toward another subtle change in bacterial metabolism upon experiencing environmental change (Figure 6C). However, our initial hypothesis was that sialic acid metabolism might be part of the strategy for *S. glacialimarina* TZS-4_T_ to survive in cold temperatures. In contrast, expression analysis of the *nanA* and *nanH* genes uncovered a statistically significant upregulation at 25°C instead of 5°C. This suggests that sialic acid metabolism might instead be linked to nutrient utilization. Phytoplankton and fungi tend to be prevalent in the Baltic Sea during summer times when the temperature is above 10°C (Kaikkonen et al., 2020). These eukaryotes have peripheral sialic acid that could act as a stimulus for sialic acid metabolising bacteria to exploit this resource. A previous study found *Shewanella* to be the third most abundant bacteria associated with *Pseudonitzschia fraudulenta* in the 2010 algal bloom in Monterey Bay, California (Sison-Mangus et al., 2016). Therefore, it is possible that certain bacterial communities possessing distinct catalytic enzymes, such as those found in *S. glacialimarina* TZS-4_T_, could have a role in degrading phytoplankton-derived organic matter.

Despite the insight that the *nanA* gene cluster is expressed in *S. glacialimarina* TZS-4_T_, we still lack a detailed understanding of the role and function of these gene products in the life cycle of the bacterium. Furthermore, the presence of some pathogenicity-associated traits, such as alpha hemolysis on blood agar, the type IV secretion system, and antibiotic resistance, may hint toward potential pathogenic properties. Notably, *S. putrefaciens* has been reported to cause disease among freshwater fish (Paździor et al., 2019) and *S. algae* is a potential human pathogen (Torri et al., 2018). However, *S. glacialimarina* TZS-4_T_ does not harm mammalians as it cannot grow above 25°C, nor does the presence of virulence-associated gene clusters constitute sufficient evidence for a potential disease-causing role in fish. Hence, further studies are needed to determine the function and utilization of these genetic elements.

Here, we have further characterized a novel cold-active *Shewanella* isolate named *S. glacialimarina* TZS-4_T_. The cold-active nature of *S. glacialimarina* TZS-4_T_ makes it an interesting model to study transcriptional and translational adaptations that facilitate metabolism at low temperatures. For instance, elucidating the RNA-based regulatory mechanisms that govern translation – such as the dynamics of post-transcriptional RNA modification, which is known to among other facilitate the translation of stress-specific gene transcripts (Koh and Sarin, 2018) – may offer further insights into the modulation of cellular responses to environmental stress. Moreover, *S. glacialimarina* TZS-4_T_ could also provide a platform to study cold-active enzymes, such as sialidase and *N*-acetylneuramidase. Consequently, based on our phylogenetic, phenotypic, and genomic characterization, we present *S. glacialimarina* TZS-4_T_ as a new species belonging to the genus *Shewanella*.

## Data Availability Statement

The data presented in the study are deposited in the GenBank repository, accession number CP041216.

## Author Contributions

MSQ and LPS conceived the study. MSQ, ML, M-MH, and BG-Z performed the experimental work. MSQ, RK, and ER analyzed the data with input from DB and LPS. MSQ wrote the first draft of the manuscript. ML, ER, and LPS edited the first draft of the manuscript. All authors contributed to the article and approved the submitted version.

## Funding

This work was supported by the Academy of Finland Academy Research Fellow program (grants #294917, #307215, and #327181 to LPS) and Academy Professor program (grants #255342, #256518, and #283072 to DB); the Novo Nordisk Foundation Emerging Investigator grant (#NNF18OC0054454 to LPS); and the Sigrid Jusélius Foundation Young Group Leader grant (to LPS). Open access fees are covered by the Helsinki University Library.

## Conflict of Interest

The authors declare that the research was conducted in the absence of any commercial or financial relationships that could be construed as a potential conflict of interest.

## Publisher’s Note

All claims expressed in this article are solely those of the authors and do not necessarily represent those of their affiliated organizations, or those of the publisher, the editors and the reviewers. Any product that may be evaluated in this article, or claim that may be made by its manufacturer, is not guaranteed or endorsed by the publisher.

## Footnotes

^1^ http://ekhidna2.biocenter.helsinki.fi/AAI/

^2^ https://eu.idtdna.com/PrimerQuest/Home/Index

^3^ https://www.sanger.ac.uk/tool/artemis/
