# Supplementary material for: Cold-Active Shewanella glacialimarina TZS-4T nov. Features a Temperature-Dependent Fatty Acid Profile and Putative Sialic Acid Metabolism
Source: Front Microbiol. 2021 Oct 1;12:737641. doi: 10.3389/fmicb.2021.737641 (PMC8519357; doi:10.3389/fmicb.2021.737641)
Supplement: Supplementary file 1 [file Data_Sheet_1.PDF]

Supplementary Material

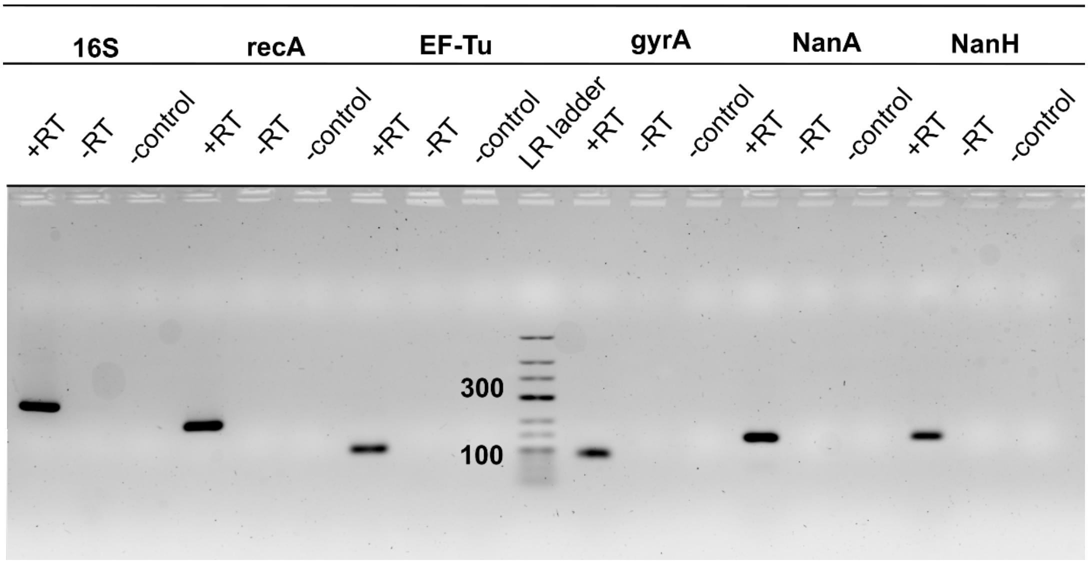

Supplementary Figure SF-1 | Agarose gel electrophoresis of cDNA amplified using primer pairs for 16S rRNA, *recA*, *EF-Tu*, *gyrA*, *nanA*, and *nanH*. Size marker (middle lane) is the low range DNA ladder SM1193 [Thermo Scientific]. Legend: (+RT), reverse transcribed cDNA; (-RT), no reverse transcription reaction; (– control), no template.

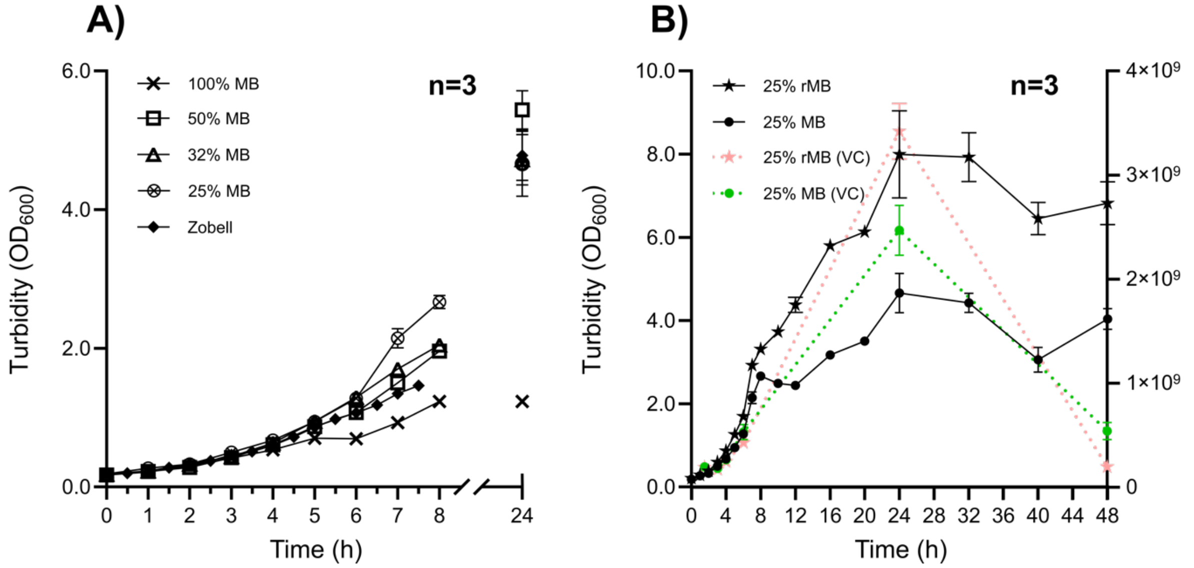

Supplementary Figure SF-2 | Optimization of growth media for *Shewanella glacialimarina* TZS-4$_T$. A) Growth of *S. glacialimarina* TZS-4$_T$ at different dilutions of marine broth (MB) and Zobell media at 15°C (started at OD_{600} 0.2). B) Two-day (48 h) growth curves at 15°C for enriched 25% MB (rMB; supplemented with yeast extract and peptone to a 1.75-fold higher concentration than in 100% MB) and 25% MB (supplemented with yeast extract and peptone to a final concentration equivalent to 100% MB). The OD_{600} measurements are depicted as solid lines and the viable counts (VC; in cfu/ml) are shown as a pink dotted line and a green dotted line for rMB and 25% MB, respectively. The cultures were started at OD_{600} 0.2.

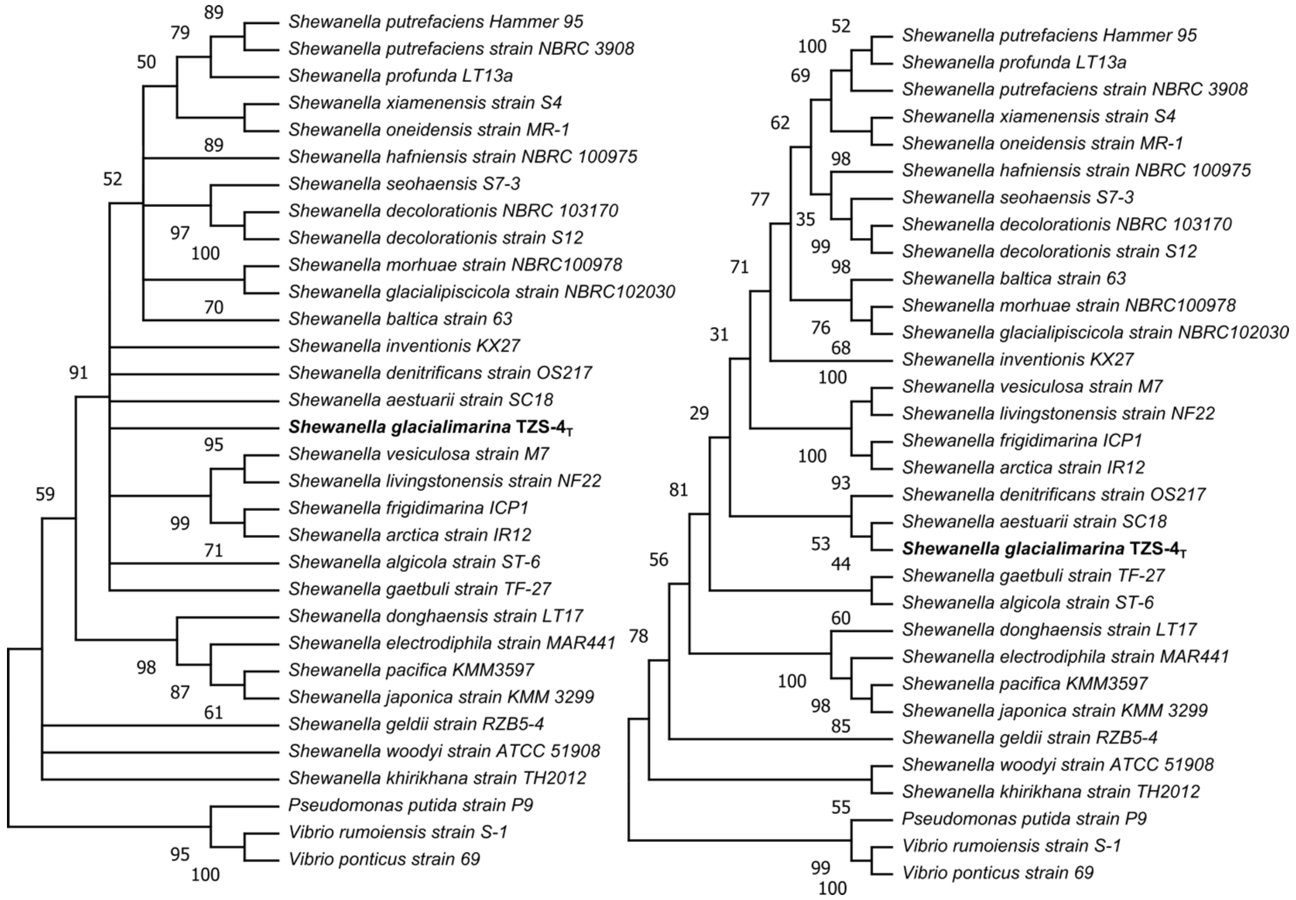

Supplementary Figure SF-3 | Phylogenetic analysis of *Shewanella glacialimarina* TZS-4$_T$ (bolded) showing maximum parsimony (left panel) and neighbor-joining (right panel) phylogenetic trees based on 16S rRNA sequences with 100 replicates as bootstrap.

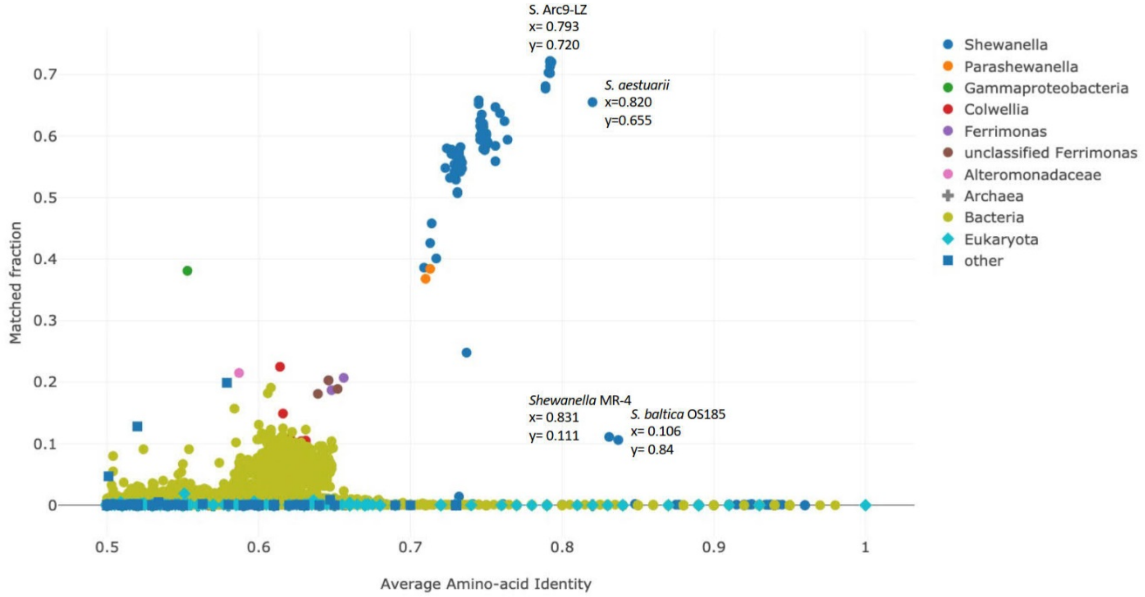

Supplementary Figure SF-4 | Average amino acid identity of *Shewanella glacialimarina* TZS-4$_T$ in comparison to other bacterial species.

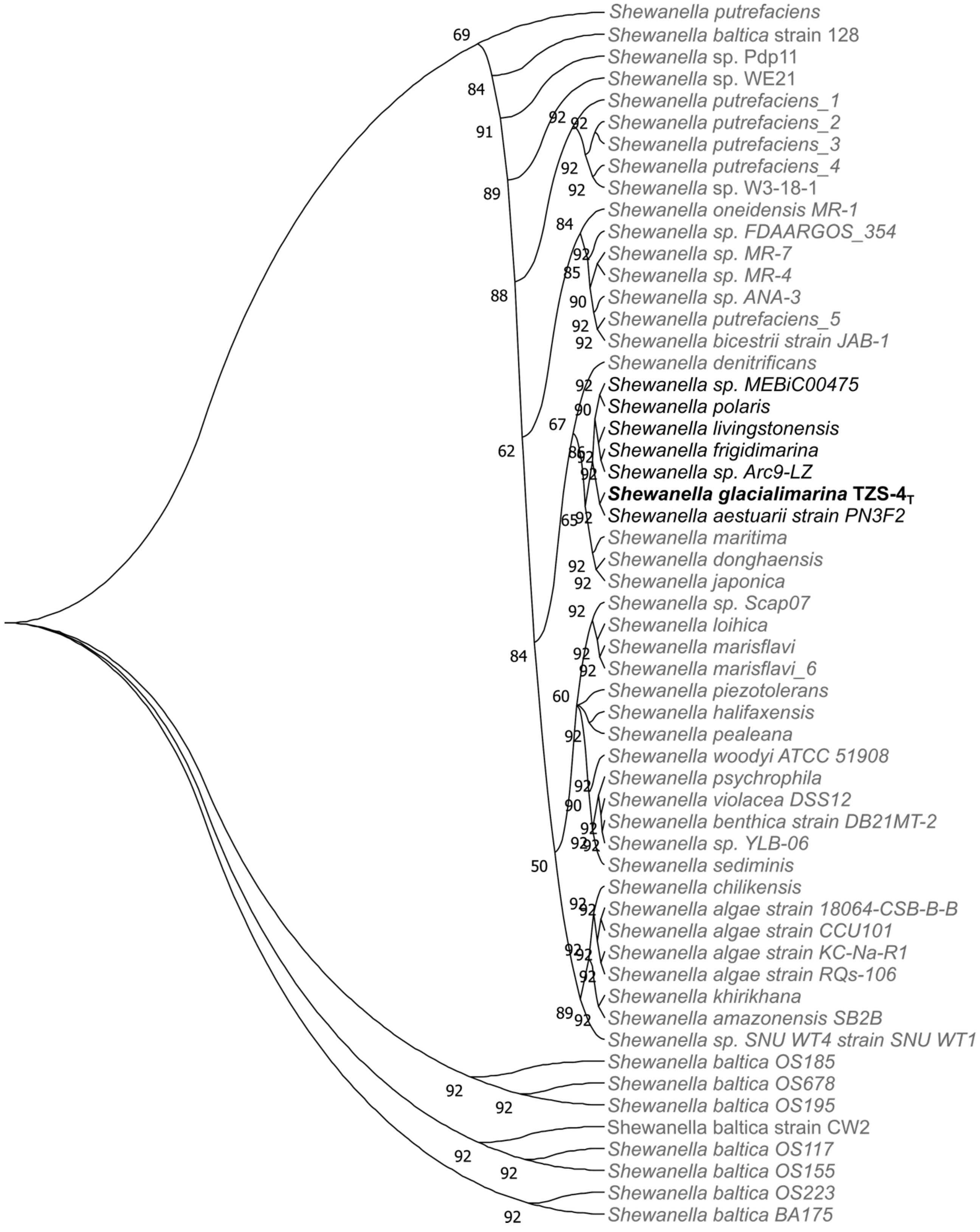

Supplementary Figure SF-5 | Phylogenetic tree based on the 92 most conserved genes (Na et al., 2018) depicting *Shewanella glacialimarina* TZS-4_T (bolded, in black) clustering with other cold-active *Shewanella* bacteria (in black). The UBCG ver 3.0 (https://www.ezbiocloud.net/tools/ubcg) pipeline was used to construct the phylogenetic tree.

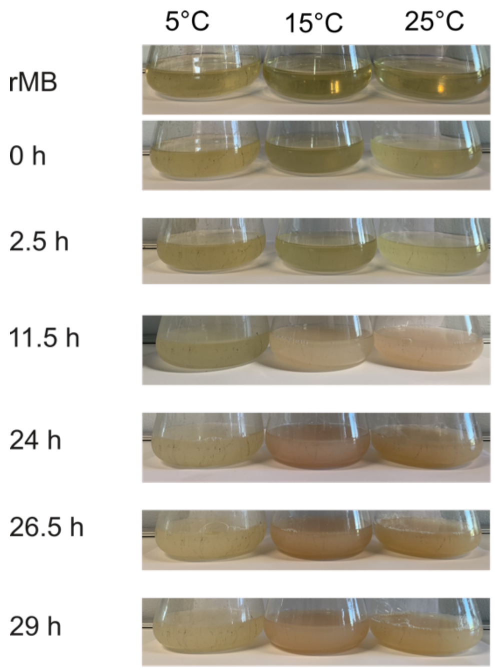

Supplementary Figure SF-6 | Pigment formation in *Shewanella glacialimarina* TZS-4$_T$ when grown for up to 29 h in rMB, shielded from light at 5°C, 15°C, and 25°C, respectively.

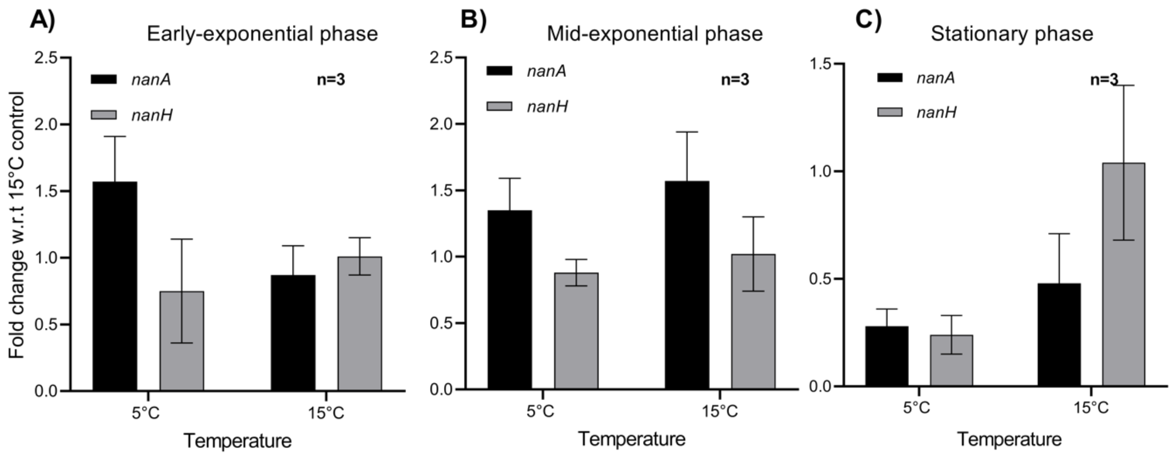

Supplementary Figure SF-7 | RT-qPCR expression analysis of sialic acid catabolic enzyme genes in *Shewanella glacialimarina* TZS-4$_T$ at the A) early exponential growth phase (O.D$_{600}$ = 0.8), B) mid-exponential growth phase (O.D$_{600}$ = 3.5), and C) stationary phase (O.D$_{600}$ = 6.0). The error bars depict standard deviation (n=3).

Supplementary Table ST-1 | List of primer sequences used in qPCR analysis.

Gene description	Gene name	Primer sequence 5´-3´	Efficiency (%)	R-squared value
Sialidase	*nanH*-forward<br>*nanH*-reverse	AGGTCCAGGTCAAGGCATTCAA<br>CCGCTATCGTCGCTAAATACAGTC	102.33	0.99
N-acetyl neuraminate lyase	*nanA*-forward<br>*nanA*-reverse	GCGTCATCCCAAATCTAGCC<br>AATCGCACCAACAGCCAATC	109.22	0.99
Gyrase A	*gyrA*-forward<br>*gyrA*-reverse	TCGAAGGCATCAGTGGTTTACG<br>CGAACATTGCATCTGAGTTTGAGC	107.73	0.99
DNA recombination/repair protein	*recA*-forward<br>*recA*-reverse	TAGAACTTCAGCGCGTTACC<br>GCCAACTGCCTGGTTATCT	112.33	0.99
rho-factor	*rho*-forward<br>*rho*-reverse	GTAAGATACGTCCGCCTAAAGAAGG<br>ACGTGAGCTTTCTGGCTTATCG	113.14	0.98

Supplementary Table ST-2 | ANI values calculated by fastANI tool.

***Shewanella* species**	**ANI score**	**Orthrologous match sequences**	**Total sequences**
Shewanella aestuarii strain PN3F2	79.8952	611	1485
Shewanella sp. Arc9-LZ	79.9951	581	1485
Shewanella livingstonensis strain LMG 19866	80.1032	558	1485
Shewanella frigidimarina NCIMB400	80.2681	554	1485
Shewanella polaris strain SM1901	79.9510	517	1485
Shewanella sp. MEBiC00475	79.7286	510	1485
Shewanella japonica strain KCTC 22435	78.5281	399	1485
Shewanella donghaensis strain LT17	78.7008	382	1485
Shewanella maritima strain D4-2	78.8848	314	1485
Shewanella denitrificans OS217	79.0134	303	1485

Supplementary Table ST-3 | Fatty acid composition (mol%) of *Shewanella glacialimarina* TZS-4$_T$ at 5°C, 15°C and 25°C.

Fatty acid	5°C	15°C	25°C
**Straight-chain saturated fatty acids:**			
**12:0**	4.50 ± 0.13	4.14 ± 0.09	3.71 ± 0.20
**13:0**	0.81± 0.05	0.71 ± 0.01	0.51 ± 0.03
**14:0**	6.99 ± 0.15	4.85 ± 0.09	4.03 ±0.04
**15:0**	1.94 ± 0.03	1.84 ± 0.03	1.26 ± 0.05
**16:0**	6.00 ± 0.34	7.12 ± 0.60	7.93 ± 0.12
**17:0**	0.12 ± 0.00	0.17 ± 0.01	0.17 ± 0.00
**18:0**	0.16 ± 0.06	0.42 ± 0.29	0.48 ± 0.02
**Branched saturated fatty acids:**			
***iso* 13:0**	13.58 ± 0.32	13.28 ± 0.12	14.79 ± 0.31
***iso* 14:0**	0.39 ± 0.02	0.31 ± 0.01	0.33 ± 0.04
***iso* 15:0**	8.91 ± 0.11	12.81 ± 0.33	16.94 ± 0.37
***anteiso* 15:0**	0.33 ± 0.02	0.57 ± 0.01	1.16 ± 0.01
***iso* 17:0**	0.14 ± 0.01	0.34 ± 0.00	0.84 ± 0.01
**Hydroxy fatty acids:**			
**3-OH 11:0**	0.13 ± 0.01	0.12 ± 0.01	0.06 ±0.01
**3-OH 12:0**	1.32 ± 0.08	1.47 ± 0.02	1.51 ± 0.11
**3-OH *iso* 13:0**	2.89 ± 0.12	3.19 ± 0.33	3.08 ± 0.11
**3-OH 13:0**	0.12 ± 0.01	0.13 ± 0.01	0.09 ± 0.09
**3-OH 14:0**	0.10± 0.01	0.13 ± 0.00	0.18 ± 0.02
**Monounsaturated fatty acids:**			
**14:1n-7**	1.89 ±0.004	1.44 ± 0.018	1.28 ± 0.025
**14:1n-5**	0.26 ± 0.038	0.19 ± 0.013	0.17 ± 0.007
**15:1n-8**	0.40 ± 0.019	0.40 ± 0.009	0.35 ± 0.011
**15:1n-6**	0.41 ± 0.038	0.42 ±0.035	0.31 ± 0.018
**16:1n-9**	0.31 ± 0.012	0.37 ± 0.009	0.39 ± 0.016
**16:1n-7**	36.59 ± 0.424	34.99 ± 0.386	31.57 ± 0.170
**16:1n-5**	0.13 ± 0.013	0.08 ± 0.038	0.08± 0.006
**17:1n-8**	0.11 ± 0.098	1.46 ± 0.028	1.16 ± 0.042
**17:1n-6**	0.28 ± 0.011	0.31 ± 0.053	0.23 ± 0.009
**18:1n-9**	0.29 ± 0.012	0.54 ± 0.015	0.73 ± 0.023
**18:1n-7**	2.64 ± 0.059	3.71 ± 0.048	4.00 ±0.016
**Polyunsaturated fatty acids:**			
**18:4n-3**	0.20 ±0.006	0.12 ± 0.004	0.07 ±0.007
**20:4n-6**	0.06 ± 0.008	0.07 ± 0.001	0.06 ± 0.001
**20:4n-3**	0.16 ± 0.003	0.10 ± 0.001	0.05 ± 0.002
**20:5n-3**	5.84 ± 0.110	3.21 ± 0.015	1.35 ± 0.017
